# Efficient Production Strategy of a Novel Postbiotic Produced by *Bacillus subtilis* and Its Antioxidant and Anti-Inflammatory Effects

**DOI:** 10.3390/molecules30102089

**Published:** 2025-05-08

**Authors:** Jing Zhang, Rijun Zhang, Junyong Wang, Zaheer Abbas, Yucui Tong, Yong Fang, Yichen Zhou, Haosen Zhang, Zhenzhen Li, Dayong Si, Xubiao Wei

**Affiliations:** State Key Laboratory of Animal Nutrition and Feeding, College of Animal Science and Technology, China Agricultural University, Beijing 100193, China; zhangjing97@cau.edu.cn (J.Z.);

**Keywords:** anti-inflammatory, antioxidant, *Bacillus subtilis*, exopolysaccharide, optimization, postbiotics

## Abstract

Microbially synthesized postbiotics have unique properties and advantages; however, systematic studies on the efficient production and biological functions of postbiotics from *Bacillus subtilis* are limited, which greatly restricts their applications. In this study, we obtained a novel crude exopolysaccharide (EPS) postbiotic from *Bacillus subtilis* H4. We systematically optimized the EPS production strategy using single-factor analysis, Plackett–Burman design, the path of steepest ascent method, and response surface methodology. The optimized EPS yield was significantly improved, with a maximum yield of 15.01 g/L under the addition of 4.12% soy peptone, 8.99% sucrose, and 0.06% MnSO_4_. We found that EPS is a neutral, heterogeneous polysaccharide with a pyranose ring, with a molecular weight of 44,304.913 kDa and a melting point of 218 °C. It consists of glucose, galactose, arabinose, glucosamine, and mannose at a molar ratio of 58.85:19.81:14.75:10.89:6.58. EPS exhibits strong antioxidant capacities, scavenging ABTS and DPPH radicals with IC50 values of 1 and 6 mg/mL, respectively. Moreover, it shows notable anti-inflammatory properties, dramatically inhibiting the lipopolysaccharide (LPS)-induced elevation of nitric oxide (NO) levels and over-activation of the TLR4-NF-κB signaling pathway. These findings highlight the potential of EPS as a multifunctional bioactive compound, offering great promise for its application in the food, clinical, and feed industries.

## 1. Introduction

Postbiotics were defined as “inanimate microorganisms and/or their components that confer a health benefit on the host” by the International Scientific Association of Probiotics and Prebiotics (ISAPP) [1]. Postbiotics are distinguished by their elemental composition, including organic acids, phenolic acids, bioactive peptides, and polysaccharides [2]. It has multiple bioactive effects, such as antioxidant, anti-inflammatory, and immunomodulatory properties [3]. Exopolysaccharides (EPS) are high molecular weight, long-chain polymers secreted into the extracellular environment by microorganisms during growth and metabolism [4,5]. As an important member of the postbiotics, EPS also has a variety of biological functions.

Although exopolysaccharides have been isolated and characterized from various types of microorganisms in the last decades, due to low yield, only a few EPSs have been used in practical production, like xanthan from *Xanthomonas campestris* and gellan from *Sphingomonas* spp., which are used as thickeners and stabilizers in the food industry [6,7]. To improve the EPS yield, the strategies primarily revolve around genetic manipulation and optimization of fermentation conditions. The genetic approaches typically involve altering the genes of enzymes critical to EPS production [8]. However, genetic approaches remain challenging due to the complexity of EPS synthesis. More advanced techniques like Plackett–Burman design (PBD) and response surface methodology (RSM) have become powerful tools for the optimization of EPS production by considering multiple factors simultaneously [9]. RSM optimization has been demonstrated to increase EPS yield by 31.32% in *Bacillus velezensis* [10]. After optimization using PBD and RSM, the EPS production from *Lactobacillus plantarum* increased from 53.34 mg/L to 97.85 mg/L, representing an 84.70% enhancement [11]. Optimizing the fermentation conditions of EPS-producing bacteria with the help of these experimental and analytical tools is important for improving EPS yield.

Oxidative stress and inflammation play a vital role in the development of many chronic diseases [12]. Oxidative stress results from the imbalance between the body’s production of reactive oxygen species (ROS) and the scavenging of ROS by the antioxidant systems [13]. Furthermore, oxidative stress and inflammation are inextricably linked and often occur simultaneously [14]. Therefore, there is an urgent need to find a substance with both antioxidant and anti-inflammatory properties. *Bacillus* species exist in various ecosystems and produce exopolysaccharides with excellent biological properties such as antioxidant, anti-inflammatory, anti-bacterial, and antiviral activities [15,16,17]. *Bacillus subtilis* is one of the known Gram-positive model organisms and is recognized as a safe strain by the Food and Drug Administration [18]. Current research on EPS of *Bacillus subtilis* is mostly focused on the production optimization, biological functions, or physicochemical properties [19,20,21]. However, systematic studies on the physicochemical properties and biological functions of EPS from *Bacillus subtilis* are limited.

In this study, the strain *Bacillus subtilis* H4 was screened from the intestine of a healthy broiler. This study aimed to exploit an efficient production optimization strategy and investigate the physicochemical properties and biological activities of novel postbiotic-crude EPS from *Bacillus subtilis* H4. The ability of *Bacillus subtilis* H4 to produce EPS was significantly enhanced by systematic optimization of fermentation conditions using Plackett–Burman and Box–Behnken design (BBD). The EPS exhibited outstanding thermostability, antioxidant, and anti-inflammatory activities. These findings provide powerful theoretical guidance and technical support for the application of EPS postbiotics in the food field, as well as their industrialization and exploitation.

## 2. Results

### 2.1. Identification of Strain H4

**Figure 1:**
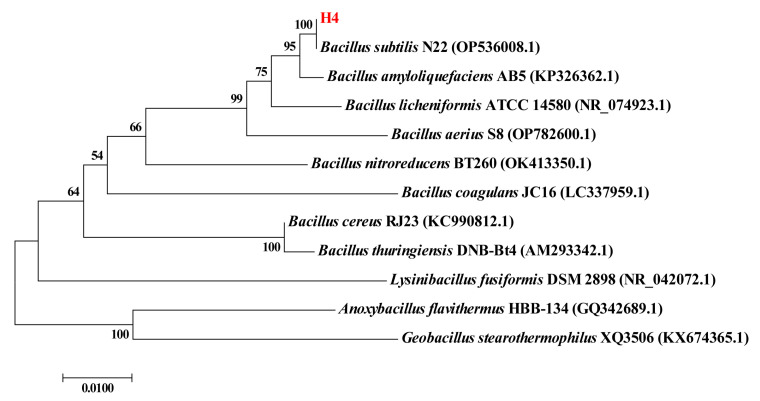
The phylogenetic tree of the strain *Bacillus subtilis* H4 using the neighbor-joining method with 1000 bootstrap replications. The scale bars represent 0.01.

The strain used in the present study was isolated from the intestine of a healthy broiler and identified by 16S rRNA sequencing. The phylogenetic tree is shown in Figure 1. The results revealed that strain H4 is closely related to *Bacillus subtilis* N22 with 100% similarity, leading to its identification as *Bacillus subtilis*.

### 2.2. Optimization Strategies of EPS Production

The main optimization strategies for EPS production include single-factor, PBD, path of steepest ascent (PSA), and BBD analytical experiments. The objective of the sequential experiments was to optimize the fermentation parameters to obtain maximum EPS yield from *Bacillus subtilis* H4.

#### 2.2.1. Single-Factor Analytical Experiment

**Figure 2:**
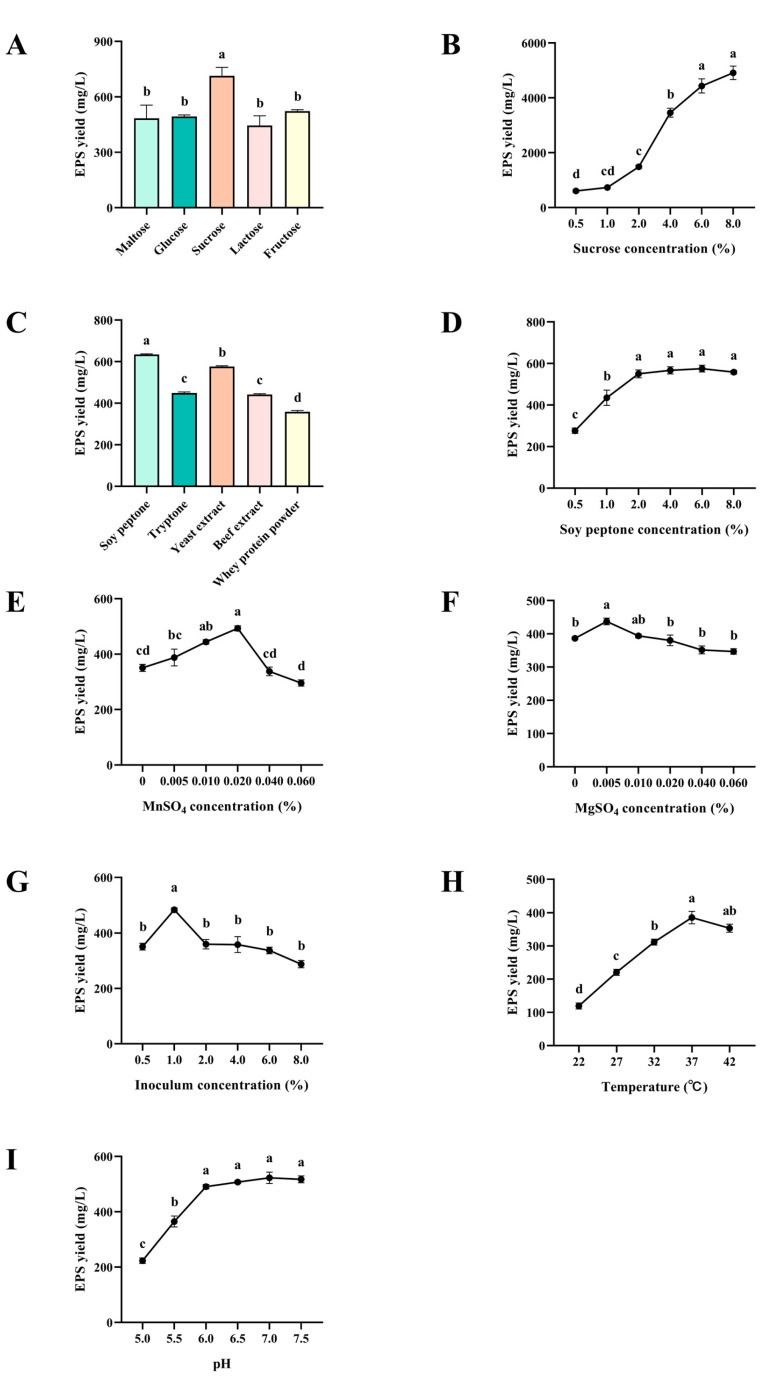
Effects of single-factor optimization on the yield of EPS from *B. subtilis* H4: (**A**) carbon sources, (**B**) sucrose concentration, (**C**) nitrogen sources, (**D**) soy peptone concentration, (**E**) MnSO_4_ concentration, (**F**) MgSO_4_ concentration, (**G**) inoculum concentration, (**H**) fermentation temperature, and (**I**) initial pH. The data were presented as mean ± SEM (*n* = 3). Different lowercase letters indicate statistical significance (*p* < 0.05).

The effects of medium composition and fermentation conditions on EPS yield are shown in Figure 2. Sucrose was the optimal carbon source, and the EPS yield increased as sucrose content increased in the range of 0.5–6% (Figure 2A,B). Among the five nitrogen sources, soy peptone had the highest EPS yield (Figure 2C). There was a significant increase in the yield of EPS as the soy peptone increased from 0.5% to 2.0% (*p* < 0.05), and then the yield of EPS reached the stationary phase with the increase of soy peptone from 2.0% to 8.0% (Figure 2D). As for metal ions, the addition of MnSO_4_ and MgSO_4_ enhanced the EPS yield. The maximum EPS yield was achieved when the concentrations of MnSO_4_ and MgSO_4_ were 0.02% (Figure 2E) and 0.005% (Figure 2F), respectively. The present study chose three fermentation conditions to optimize the EPS yield. The optimal inoculum concentration and temperature for EPS yield were 1% and 37 °C, respectively (Figure 2G,H). It was noteworthy that the highest EPS yield was achieved at a wide range of pH from 6.0 to 7.5 (Figure 2I).

#### 2.2.2. Determination of Significant Factors Influencing EPS Yield via PBD

**Figure 3:**
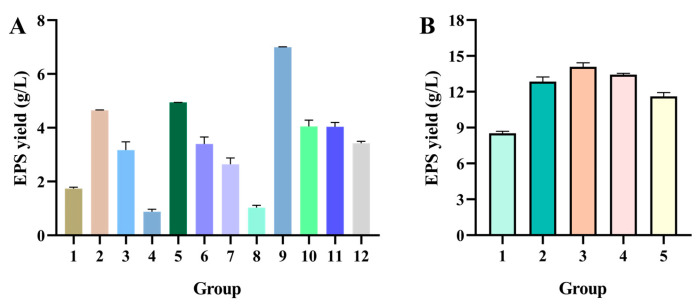
The EPS yield of (**A**) PB and (**B**) PSA experiments. The data were presented as mean ± SEM (*n* = 3). The details of groups of PB and PSA experiments are presented in Tables 6 and 7.

The EPS yield of the 12 groups designed by PBD is presented in Figure 3A. The ANOVA details of the fitted equation for the EPS yield are displayed in Table 1. Among the seven factors, concentrations of sucrose, soy peptone, and MnSO_4_ significantly influenced the EPS yield (*p* < 0.05). All three factors had a positive correlation with the EPS yield. The experimental data were subjected to multiple regression analysis and a significance test, which revealed the following functional relationship between the factors and the response values:

Y (g/L) = 3.41 + 0.59 A + 0.93 B + 1.02 C + 0.044 D − 0.34 E − 0.28 F − 0.35 G(1)

where Y represents EPS yield and A-G represent the coded value of sucrose concentration, soy peptone concentration, MnSO_4_ concentration, MgSO_4_ concentration, inoculum concentration, temperature, and initial pH, respectively.

#### 2.2.3. Path of Steepest Ascent Determined the Center of Response Surface

The step sizes of soy peptone (X_1_), sucrose (X_2_), and MnSO_4_ (X_3_) were 1.5%, 1.5%, and 0.015%, respectively. Group 3, with the addition of 4.5% soy peptone, 8.5% sucrose, and 0.055% MnSO_4_, exhibited the highest EPS yield (Figure 3B). The above addition parameters were set as the center point of the BBD experiment to further optimize the EPS yield.

#### 2.2.4. Box–Behnken Elucidated the Interactions Between Various Factors

The experimental design and the response values for EPS yields are presented in Table 2. A total of 17 groups containing 5 central points were designed using a Box–Behnken. Based on the results, quadratic multinomial regression equations between EPS yield and factors were established.

Y (g/L) = 14.07 − 0.49 X_1_ + 1.19 X_2_ + 2.40 X_3_ − 1.72 X_1_X_2_− 0.46 X_1_X_3_ + 1.24 X_2_X_3_ − 2.57 X_1_^2^ − 3.44 X_2_^2^ − 2.85 X_3_^2^ (2)

where Y represented EPS yield and X_1_, X_2_, and X_3_ represented the coded values of soy peptone, sucrose, and MnSO_4_, respectively.

The ANOVA analysis of the response surface model is displayed in Table 3. The *p* value of the model was less than 0.001, which implied that the model was enormously significant. Two linear coefficients of X_2_ and X_3,_ and two interactive coefficients of X_1_X_2_ and X_2_X_3_ significantly affected the EPS yield. The regression model fitted the functional relationship between the test variables and the response values, indicating that the model could accurately predict EPS yield (*p* value of no fit > 0.05).

**Figure 4:**
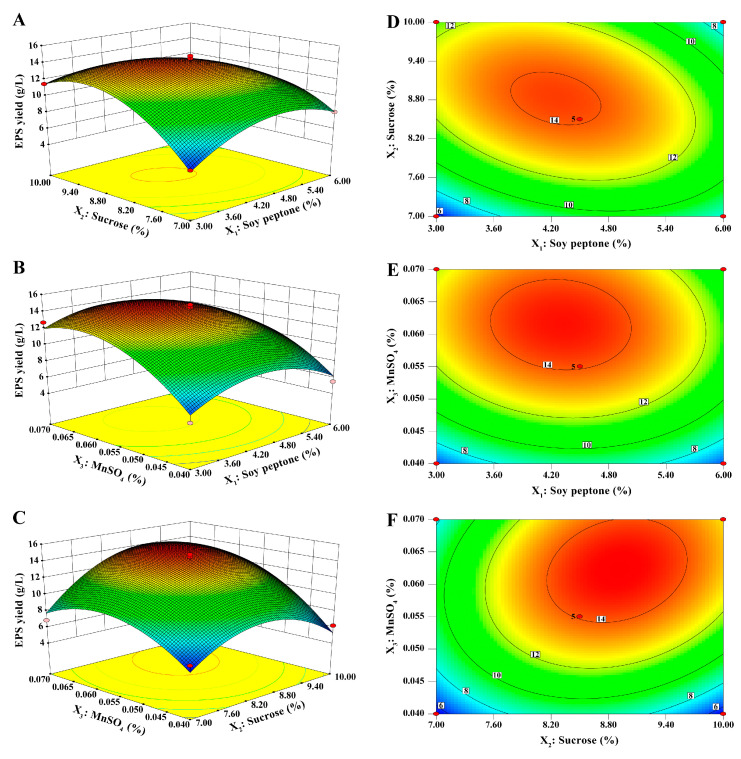
Three-dimensional response surface plots and contour plots exhibited the interactive effects of the (**A**,**D**) soy peptone and sucrose concentration, (**B**,**E**) soy peptone and MnSO_4_ concentration, and (**C**,**F**) sucrose and MnSO_4_ concentration on EPS yield.

The 3D response surface plots and contour plots of soy peptone, sucrose, and MnSO_4_ based on the regression equation are displayed in Figure 4. They visualized the relationship between EPS yield and the levels of each variable, as well as the interaction between the two test factors. Figure 4A,D represents the interaction effect of soy peptone and sucrose on the EPS yield. At 0.055% addition of MnSO_4_, the EPS yield increased and then decreased with the increase of soy peptone and sucrose concentration. The other groups (soy peptone and MnSO_4_, Figure 4B,E, and sucrose and MnSO_4_, Figure 4C,F) showed the same trend. The EPS yield tended to increase as any two factors were enhanced, and then decreased after the interaction between the two variables reached their maximum value. After optimization by the BBD experiment, the best fermentation conditions for EPS production were obtained as follows: 4.12% soy peptone, 8.99% sucrose, and 0.06% MnSO_4_. To confirm the true reliability of the estimated values, the experiment was repeated three times using the optimized additions generated by the BBD model. The average EPS yield measured was 15.01 g/L, which was close to the predicted value (14.94 g/L). It demonstrated that the model is reliable and the EPS yield of *B. subtilis* H4 could be predicted by the regression equation.

After optimization by the BBD experiment, the best fermentation conditions for EPS production were obtained as follows: 4.12% soy peptone, 8.99% sucrose, and 0.06% MnSO_4_. To confirm the true reliability of the estimated values, the experiment was repeated three times using the optimized additions generated by the BBD model. The average EPS yield measured was 15.01 g/L, which was close to the predicted value (14.94 g/L). It demonstrated that the model is reliable and the EPS yield of *B. subtilis* H4 could be predicted by the regression equation.

### 2.3. Chemical Composition of EPS

Chemical composition analysis revealed that EPS consisted of total carbohydrate (85.37%) and protein (0.25%), and lacked glucuronic acid, reducing sugar, and sulfate (Table 4).

### 2.4. Molecular Weight and Monosaccharide Compositions

**Figure 5:**
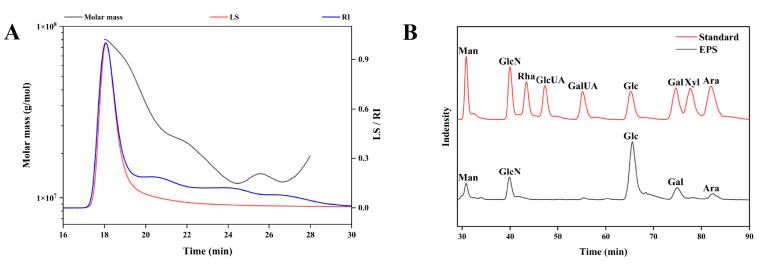
(**A**) HPGPC chromatogram of the molecular weight of EPS. (**B**) HPLC chromatogram of the monosaccharide composition of EPS.

High-performance gel permeation chromatography (HPGPC) map of EPS displayed one primary peak and three smaller peaks, indicating the presence of four polysaccharides (Figure 5A). The weight-average molecular weight (Mw) of the primary EPS was 44,304.913 kDa, the number-average molecular weight (Mn) was 26,713.583 kDa, and the polydispersity index (Mw/Mn) was 1.659. High-performance liquid chromatography (HPLC) analysis of the monosaccharide composition showed the presence of glucose, galactose, arabinose, glucosamine, and mannose in the EPS with a molar ratio of 58.85:19.81:14.75:10.89:6.58. As shown in Figure 5B, EPS consisted mainly of glucose and lacked glucuronic acid, indicating that EPS was a neutral polysaccharide.

### 2.5. Fourier Transform Infrared Spectroscopy (FTIR) Spectrum

**Figure 6:**
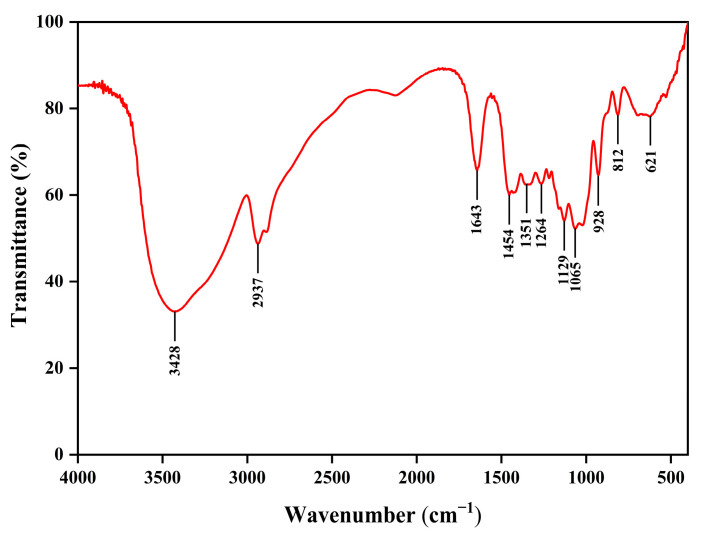
FTIR spectrum of EPS.

EPS displayed characteristic absorption peaks of polysaccharides analyzed by FTIR (Figure 6). The intermolecular O-H stretching vibration of hydroxyl groups was relevant to the broad and intense band at 3428 cm^−1^. The sharp peak at 2937 cm^−1^ corresponded to the C-H stretching vibration of methyl or methylene groups. A sharp and strong carbonyl stretching vibration was observed at 1643 cm^−1^. The weak peaks at 1454 and 1351 cm^−1^ were associated with the bending and stretching vibrations of C-H, respectively. The peak at 1264 cm^−1^ indicated C-O-C stretching vibration. The peaks at 1065 cm^−1^ and 1021 cm^−1^ were the characteristic peaks of the pyranose ring structure, suggesting the presence of pyran glycosidic ring C-OH in EPS. In addition, the weak peaks at 928 cm^−1^ and 812 cm^−1^ confirmed the co-existence of α- and β-glycosidic bonds in EPS (Figure 6).

### 2.6. EPS Morphological Structure

**Figure 7:**
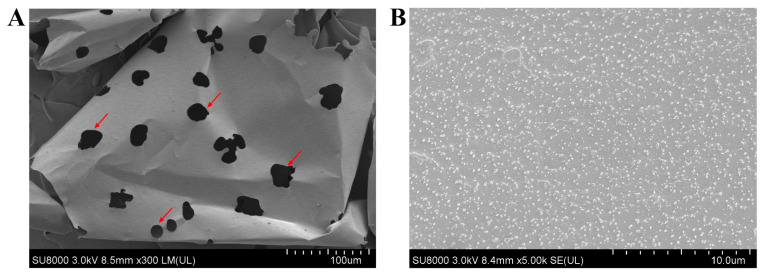
Morphological structure of EPS by scanning electron microscopy (**A**), 300× magnification, (**B**), 5000× magnification. The holes were labeled with red arrows.

The morphological structure of EPS was observed by scanning electron microscopy. As shown in Figure 7A, EPS displayed a flaky morphology with a smooth surface, interspersed with holes of heterogeneous sizes (12–36 μm) and irregular distribution. Upon further magnification, we observed that the EPS surface was dense and covered with small particles (Figure 7B).

### 2.7. Thermal Properties of EPS

**Figure 8:**
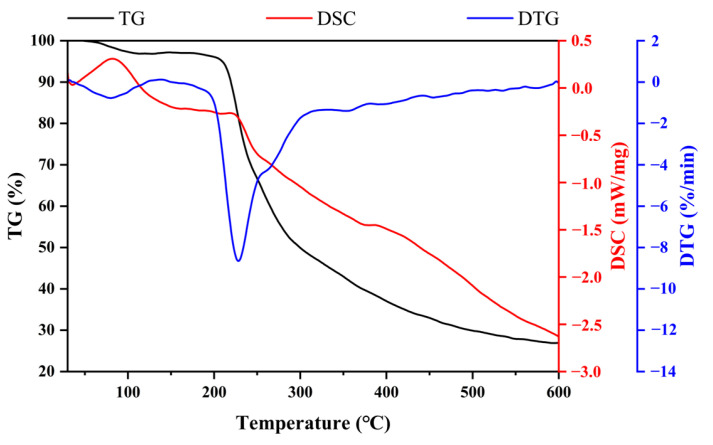
Thermal properties of EPS analyzed by TG, DTG, and DSC analyses.

The thermogravimetric (TG) and differential thermogravimetric (DTG) curves of EPS are presented in Figure 8. The TG curve revealed the weight loss of EPS during heating from 30 °C to 600 °C and showed three diverse stages. In the first stage (30–200 °C), the mass decreased by 4% due to the vaporization of free and bound water of EPS. Subsequently, a dramatic decrease of 44% was observed from 200 to 292.51 °C. The second stage was primarily attributed to the pyrolysis of EPS. The mass decreased slowly and reached a plateau in the third stage. About 27% of the EPS residue was obtained at 600 °C. The DTG curve of EPS showed two peaks at 80 °C and 228 °C, where the weight of EPS decreased rapidly. Differential scanning calorimetry (DSC) was conducted to identify the existence of endothermal or exothermal changes with the increase of temperature. The DSC curve of EPS showed endothermic peaks at 82 °C and 218 °C without an exothermal peak. The melting point of EPS was 218 °C.

### 2.8. Antioxidant Capacity of EPS

**Figure 9:**
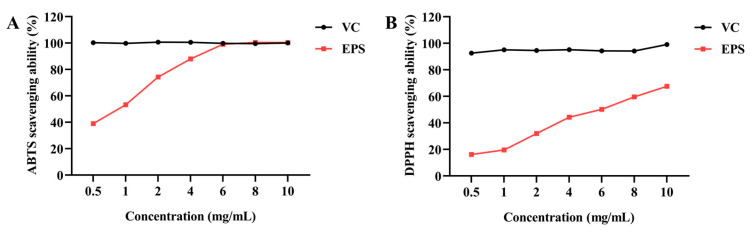
Antioxidant capacity of EPS in vitro. (**A**) ABTS and (**B**) DPPH radical scavenging capacities of EPS. VC was set as a positive control. The data were presented as mean ± SEM (*n* = 3).

The antioxidant capacity of EPS was evaluated based on the ABTS and DPPH radical scavenging assays. As depicted in Figure 9, EPS scavenges ABTS and DPPH radicals in a dose-dependent manner. The IC50 of EPS to scavenge ABTS and DPPH radicals was 1 and 6 mg/mL, respectively. EPS showed a similar ABTS scavenging ability to ascorbic acid (VC) when the EPS concentration was 6 mg/mL.

### 2.9. Effects of EPS on the Expression of Pro-Inflammatory Cytokines

**Figure 10:**
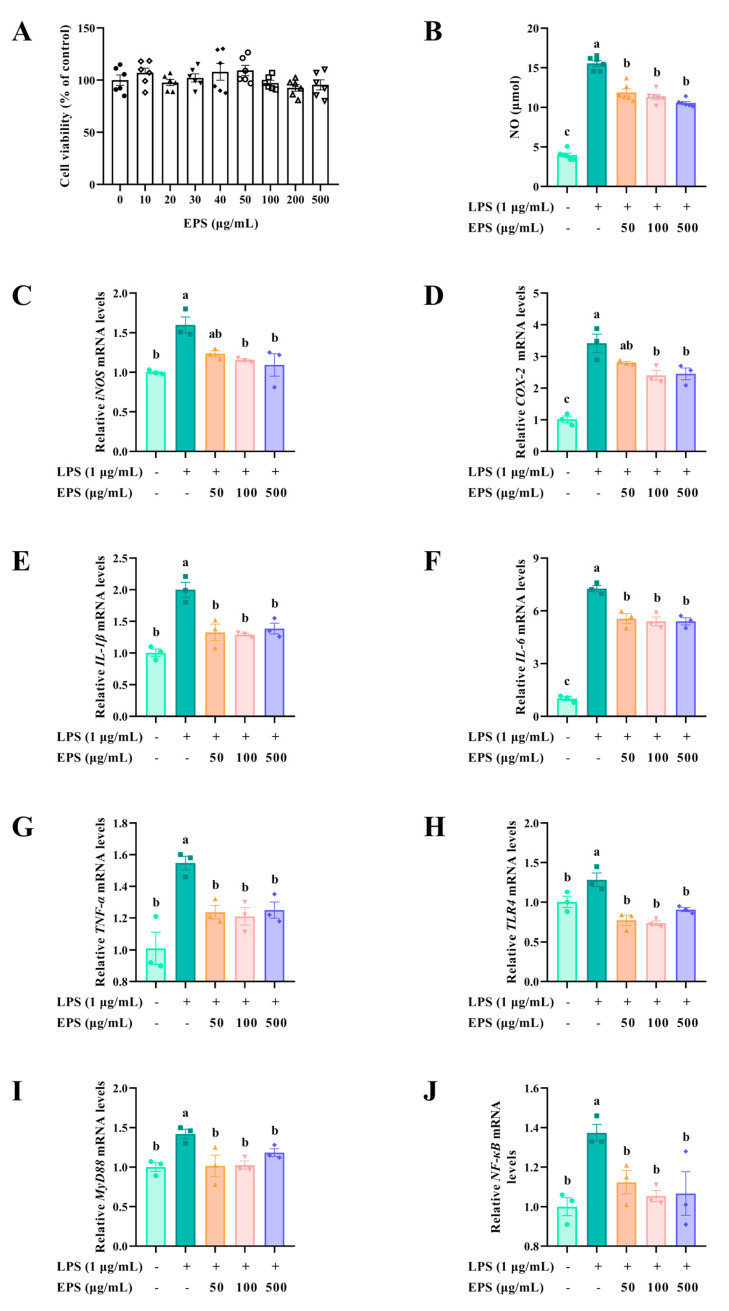
EPS diminished the expression levels of pro-inflammatory cytokines in LPS-induced inflammation. (**A**) Cell viability of EPS on the RAW264.7 cells (*n* = 6). (**B**) EPS reduced the NO production (*n* = 6). EPS decreased the expression levels of pro-inflammatory cytokines (**C**) *iNOS*, (**D**) *COX-2*, (**E**) *IL-1β*, (**F**) *IL-6*, and (**G**) *TNF-α*, and TLR4-NF-κB signaling pathway proteins (**H**) *TLR4*, (**I**) *MyD88*, and (**J**) *NF-κB* (*n* = 3). The data were presented as mean ± SEM. Different lowercase letters indicate significant differences (*p* < 0.05).

The effect of EPS on RAW264.7 cell viability is depicted in Figure 10A. When the concentration of EPS was within 500 μg/mL, it was nontoxic to the viability of RAW264.7 cells. Subsequently, NO, a crucial inflammatory biomarker, was measured. The content of NO was dramatically increased when treated with LPS (*p* < 0.05). Treating RAW264.7 with EPS reduced the production of NO (Figure 10B).

The expression levels of NO-producing enzyme *iNOS*, prostaglandin E2-producing enzyme *COX-2*, pro-inflammatory cytokines *IL-1β*, *IL-6*, and *TNF-α,* and proteins of the TLR4-NF-κB signaling pathway were further determined by qPCR. LPS remarkably elevated the expression levels of *iNOS* and *COX-2* (*p* < 0.05). The addition of EPS alleviated LPS-induced abnormal elevation of *iNOS* and *COX-2* levels (Figure 10C,D). Pro-inflammatory cytokines showed similar results. LPS induced overexpression of *IL-1β*, *IL-6,* and *TNF-α*, whereas EPS reversed this adverse effect (Figure 10E–G). Furthermore, LPS significantly increased the expression levels of *TLR4*, *MyD88*, and *NF-κB*, and the addition of EPS restored the elevated levels to normal (*p* < 0.05) (Figure 10H–J).

## 3. Discussion

Several EPSs exhibited multiple biological abilities, while the low EPS yield extremely restrained the application of EPS in practical production. EPS from *Lacticaseibacillus rhamnosus* ACS5 had strong antioxidant and antidiabetic activities, but the crude EPS yield was 16.91 mg/L, which was far from the demand in production [22]. It’s urgent to explore an optimization strategy for the fermentation conditions. Traditional optimization methods, such as the one-factor-at-a-time method, focus on individual variables without considering their interactions. The two-step approach (PBD screening of key factors and BBD assessment of their individual and interactive effects) has become an excellent method for fermentation optimization [9]. In this study, EPS production was optimized by using a combination of single-factor analysis, PBD, PSA, and BBD. The EPS yield of *B. subtilis* was remarkably elevated after single-factor optimization. During the fermentation, the carbon source played a crucial role in the production of EPS as a substrate for EPS. Among the carbon sources tested, sucrose proved to be the most productive in increasing the EPS yield, which is in agreement with previous findings [10,23,24]. In addition to these conventional nutrients, metal ions such as Mg^2+^ and Mn^2+^ were found to play a pivotal role in EPS synthesis, likely by promoting microbial growth and metabolism [25]. MnSO_4_ was more effective than MgSO_4_, which may contribute to its usage as a co-factor or catalyst in microbial fermentation processes. To maximize the EPS yield, PBD combined with the BBD method was conducted to further improve the parameters. RSM optimization has proven to be 1.675 times more effective than one-factor optimization [26]. In the present study, the combined optimization strategies resulted in an EPS yield of 15.01 g/L, which was a significant increase from the initial 5.32 g/L and exceeded the yields of other *Bacillus* strains, such as *Bacillus* spp. NRC5 (5.2 g/L) [27], *Bacillus subtilis* (0.6 g/L) [28], *Bacillus circulans* (1.09 g/L) [26], and *Bacillus velezensis* (1.97 g/L) [10]. Although the EPS yield was significantly improved after an efficient optimization strategy, the optimization in the study was at the laboratory level, and it should be further optimized at the fermenter level in order to facilitate its industrial production. Besides, high costs are a crucial constraint in industrialized production. Finding economical raw materials like molasses, agricultural waste, and industrial by-products to replace expensive sugars and peptones is necessary to reduce costs. The utilization of *Jerusalem artichoke* and date seeds powder as carbon sources for EPS production by *Bacillus* sp. significantly reduced fermentation costs [28,29]. The fermentation optimization strategies solve the problem of low EPS yield and provide a theoretical basis for its industrialization.

In this study, we preliminarily evaluated the basic properties of EPS, a novel postbiotic from *Bacillus subtilis* H4. EPS consisted of 85.37% total carbohydrate and 0.25% protein. The content of the total carbohydrate of EPS was higher than that of EPS from *Bacillus* sp. BS11 (49.5%) [30] and *Streptomyces violaceus* (61.4%) [31], but lower than EPS from *Naematelia aurantialba* (91.03%) [32]. Due to the complexity of the chemical composition of EPS, it is difficult to characterize each chemical component. Further investigations are necessary to elucidate the unknown chemical composition of EPS. EPS molecular weights vary among species. The molecular weight of the primary EPS in the study was 44,304.913 kDa, which is comparable to that of the EPS from *Bacillus licheniformis* (44,565 kDa) [33] and *Bacillus paralicheniformis* (55,170 kDa) [34], and much larger than that of the EPS from *Bacillus* spp. (89 kDa) [35] and *Bacillus amyloliquefaciens* (8 kDa) [17]. EPS consisted of galactose, glucose, glucosamine, arabinose, and mannose, and was free of uronic acid, which was in accordance with the results of the chemical composition of EPS. EPS monosaccharides were composed of all pyranose rings except mannose. These findings were confirmed by FTIR spectroscopy, which found the featured peaks of the pyranose ring at 1065 cm^−1^ and 1021 cm^−1^. Previous studies have suggested that glucose and mannose form the core structure of water-soluble polysaccharide chains [36,37,38,39]. SEM results of EPS revealed a flaky, porous surface morphology, which is commonly observed in polysaccharides, and likely contributes to their excellent water solubility [39,40]. The thermal stability of EPS is crucial for its industrial applications. EPS from *B. subtilis* exhibited great thermostability with a degradation temperature of 228 °C. The temperature was higher than that of EPS from *Bacillus enclensis* at 130.5 °C [41] and *Bacillus megaterium* at 168 °C [42], but lower than EPS from *Bacillus tequilensis* FR9 at 239.72 °C [43] and *Bacillus velezensis* at 261.63 °C [44]. Even though these bacteria belong to the same genus, the thermal stability of EPS is highly variable. DSC determined the change in thermodynamic properties to evaluate the physical or chemical change process [23]. The DSC curve of EPS exhibited an endothermic peak of EPS with a melting point of 218 °C. Both TGA and DSC confirmed that EPS has excellent thermostability and can be safely used in different industries with high thermal processing requirements.

Subsequently, the biological capacities of EPS were evaluated. These facilitated our in-depth understanding of the relationship between the structure and function of EPS. The antioxidant capacities of EPS were assessed through ABTS and DPPH radical scavenging tests, with IC50 values of 1 mg/mL and 6 mg/mL, respectively. These results were consistent with previous findings demonstrating the antioxidant potential of EPS from *B. tequilensis* FR9 and *Bacillus* spp. NRC5 [27,43]. The ABTS and DPPH radical scavenging capacities of EPS were higher than L-EPS-ACS5 from *Lacticaseibacillus rhamnosus* ACS5, but lower than the EPS from *Lactobacillus plantarum* R301 [11,22]. This study assessed the capacity of EPS to scavenge free radicals; further studies are needed to elucidate the functional mechanism of its antioxidant activity both in vivo and in vitro.

In addition to its antioxidant effects, EPS exhibited significant anti-inflammatory properties. LPS, an endotoxin in the cell wall of Gram-negative bacteria, triggers inflammatory responses and promotes the expression of inflammatory cytokines [40]. NO, an important biomarker of the inflammatory response, is primarily catalyzed by inducible nitric oxide synthase, and plays a vital role in many inflammatory diseases [45]. In addition, prostaglandin E2 is also a vital inflammatory mediator induced by COX-2, which is usually absent or expressed at low levels in normal conditions but is overexpressed in inflammatory states [46]. In the present study, we found that EPS from *B. subtilis* H4 has excellent anti-inflammatory activity by inhibiting LPS-induced NO release and reducing the expression of proinflammatory markers such as iNOS and COX-2.

LPS is recognized by toll-like receptor 4 and signals into cells in a MyD88-dependent manner [47,48]. Increased expression of MyD88 triggers the following inflammatory cascade in NF-κB and stimulates the secretion of downstream pro-inflammatory cytokines, such as IL-1β, IL-6, and TNF-α [49,50]. The results manifested that EPS significantly inhibited the expression of IL-1β, IL-6, and TNF-α. Similarly, EPSs from *Bacillus* spp., *Bacillus thuringiensis*, and other microbial sources like *Lactobacillus plantarum*, *Limosilactobacillus fermentum*, and *Leuconostoc mesenteroides* inhibited the LPS-induced release of pro-inflammatory mediators, such as NO, IL-6, and TNF-α [11,51,52,53,54]. The inhibitory effect of EPS in this study on the mRNA levels of pro-inflammatory cytokines was stronger than that of EPS from *Lactobacillus plantarum* and *Limosilactobacillus fermentum* [11,54]. The inflammatory mediators were reduced, which revealed that the activity of signaling pathways related to inflammation was suppressed. Our results demonstrated that EPS restored LPS-induced elevated levels of TLR4, MyD88, and NF-κB to normal levels. EPS exhibited anti-inflammatory effects via suppression of the TLR4/MyD88/NF-κB pathway, consistent with studies on EPS from *Cordyceps sinensis* [55] and *Crypthecodinium cohnii* [56]. EPS is widely used in the food industry due to its functional properties, including its ability to act as a thickener, stabilizer, emulsifier, and gelling agent. In the present study, the thermostability enhanced its suitability for food applications. Taken together, these findings suggest that EPS is a promising new antioxidant and anti-inflammatory agent, laying a solid foundation for its further application in the food, pharmaceutical, and feed industries.

## 4. Materials and Methods

### 4.1. Materials

The strain *B. subtilis* H4 was isolated from the intestine of a healthy broiler, and subsequently stored in the China General Microbiological Culture Collection Center (CGMCC No. 20463). Then the strain H4 was identified by 16S rRNA sequencing. Soy peptone, beef extract, and whey protein powder were acquired from Aobox Biotechnology Co., Ltd. (Beijing, China). Maltose, glucose, sucrose, lactose, fructose, and other salts were purchased from Sinopharm Chemical Reagent Co., Ltd. (Shanghai, China). Tryptone and yeast extract, monosaccharide standards, 1-phenyl-3-methyl-5-pyrazolone (PMP), and acetonitrile (HPLC grade) were obtained from Sigma-Aldrich (St. Louis, MO, USA). LPS, 2,2′-azino-bis-3-ethylbenzothiazoline-6-sulfonic acid (ABTS), 1,1-diphenyl-2-picrylhydrazyl (DPPH), dialysis bag, penicillin, streptomycin, and cell counting kit-8 were provided by Solarbio Science &Technology Co., Ltd. (Beijing, China). Dulbecco’s Modified Eagle Medium (DMEM) was acquired from Thermo Fisher Scientific Inc. (Waltham, MA, USA). Fetal bovine serum (FBS) was purchased from Wuhan Procell Life Technology Co., Ltd. (Wuhan, China). All reagents were analytical grade unless otherwise specified.

The basic fermentation medium contained (per liter distilled water) 5.0 g of yeast extract, 10.0 g of tryptone, 10.0 g of glucose, 1.5 g of NaH_2_PO_4_, and 1.5 g of Na_2_HPO_4_.

### 4.2. Preparation of Postbiotics from B. Subtilis H4

The preserved strain *B. subtilis* H4 was streaked on a fresh beef peptone yeast (BPY) plate to obtain a single colony. The isolates were subsequently inoculated in the BPY medium at 37 °C overnight to activate the strain. Finally, the seed culture was incubated in the basic fermentation medium at 37 °C for 36 h. The fermented medium was heated at 100 °C for 15 min to inactivate the EPS-degrading enzymes. After centrifuging at 12,000 rpm for 20 min, the supernatant containing postbiotics was obtained.

### 4.3. Separation and Measurement of EPS

EPS was extracted and purified from the supernatant using the procedure provided by Sathishkumar et al. [57] with some adjustments. To precipitate the protein, 80% (*w*/*v*) trichloroacetic acid was mixed with 2 mL of supernatant and incubated at 4 °C for 8 h. Following the centrifugation at 12,000 rpm for 15 min, the supernatant was mixed with prechilled absolute ethanol (1:3, *v*/*v*) and incubated at 4 °C for 12 h to precipitate EPS. After precipitation, EPS was collected by centrifuging at 12,000 rpm for 15 min and then dissolved in 2 mL of distilled water. Finally, EPS was dialyzed in distilled water using a dialysis bag (8000–14,000 Da) at 4 °C for 24 h to remove monosaccharides. The rest of the EPS was lyophilized to explore the antioxidant and anti-inflammation activities.

The EPS content was measured using the phenol-sulfate acid method [58]. Briefly, glucose was used as a standard, and 1 mL of glucose with different concentrations ranging from 0 to 1000 μg/mL was added to 1 mL of 6% (*w*/*v*) phenol solution, respectively. Then, 5 mL concentrated sulfuric acid was blended into the above solution and stirred. Following a half-hour incubation at room temperature, the mixture’s absorbance was assayed at 490 nm. Similarly, the 1 mL dialyzed EPS was treated according to the above procedures, and the yield was calculated using the glucose standard curve (absorbance value vs. concentration).

### 4.4. Optimization Strategies for EPS Production

#### 4.4.1. Single-Factor Experiment

A preliminary investigation of the various factors affecting the EPS yield was conducted using the single-factor optimization approach. The following EPS fermentation parameters were evaluated: carbon source (maltose, glucose, sucrose, lactose, and fructose), sucrose concentration (0.5, 1.0, 2.0, 4.0, 6.0, and 8.0%), nitrogen source (tryptone, yeast extract, soy peptone, beef extract, and whey protein powder), soy peptone concentration (0.5, 1.0, 2.0, 4.0, 6.0, and 8.0%), MnSO_4_ concentration (0, 0.005, 0.010, 0.020, 0.040, and 0.060%), MgSO_4_ concentration (0, 0.005, 0.010, 0.020, 0.040, and 0.060%), inoculum concentration (0.5, 1.0, 2.0, 4.0, 6.0, and 8.0%), fermentation temperature (22, 27, 32, 37, and 42 °C) and pH (5.0, 5.5, 6.0, 6.5, 7.0, and 7.5). The EPS yield was measured as described in Section 4.3.

#### 4.4.2. Selection of Critical Factors Affected the EPS Yield Using PBD

Considering the results of the one-factor tests, the PBD was used to screen the primary factors impacting the crude EPS production from the concentration of (A) sucrose, (B) soy peptone, (C) MnSO_4_, and (D) MgSO_4_, (E) inoculum concentration, (F) temperature, and (G) initial pH. Each factor set a high level (+1) and a low level (−1), as displayed in Table 5. PBD was conducted by a design expert, and the experimental design is displayed in Table 6.

**Table 5:** Factors and corresponding levels of Plackett–Burman design.

Factors	Level	
	−1	+1
A-Sucrose (%)	4	8
B-Soy peptone (%)	1	2
C-MnSO4 (%)	0.01	0.04
D-MgSO4 (%)	0.005	0.020
E-Inoculum concentration (%)	0.5	2
F-Temperature (°C)	27	37
G-pH	5.5	6.5

**Table 6:** Experimental design of Plackett–Burman experiment.

Run	A-Sucrose	B-Soy Peptone	C-MnSO_4_	D-MgSO_4_	E-Inoculum Concentration	F-Temperature	G-pH
1	−1	−1	−1	−1	1	−1	−1
2	1	−1	1	1	−1	1	−1
3	−1	−1	1	−1	−1	1	−1
4	1	−1	−1	−1	1	1	1
5	−1	1	1	1	1	−1	−1
6	−1	1	1	−1	1	1	1
7	−1	1	−1	1	−1	1	1
8	−1	−1	−1	1	−1	−1	1
9	1	1	1	−1	−1	−1	1
10	1	1	−1	1	1	1	−1
11	1	1	−1	−1	−1	−1	-1
12	1	-1	1	1	1	-1	1

#### 4.4.3. Path of Steepest Ascent Method

The dominant factors and the direction of the movement were determined by PBD. Subsequently, the PSA method was used to confirm the center point of the response surface. The increments of three significant factors included soy peptone, sucrose, and MnSO_4_ were 1.5%, 1.5%, and 0.15%, respectively. The details of the experimental design are presented in Table 7.

**Table 7:** Experimental design of the path of steepest ascent experiment.

Run	X_1_-Soy Peptone (%)	X_2_-Sucrose (%)	X_3_-MnSO_4_ (%)
1	1.5	5.5	0.025
2	3.0	7.0	0.040
3	4.5	8.5	0.055
4	6.0	10.0	0.070
5	7.5	11.5	0.085

#### 4.4.4. Response Surface Methodology

The relationship between the response values and different variables was determined using RSM. According to the results of the steepest ascent path, a BBD was conducted to further optimize the fermentation parameters of factors (soy peptone, sucrose, and MnSO_4_). As shown in Table 8, a three-variable and three-level (−1, 0, and +1) response surface design with 17 runs was generated by Design-Expert software 8.0.6. The levels were set by the PSA experiment.

### 4.5. Chemical Composition of EPS

One milligram of EPS was dissolved in 1 mL of H_2_O. The concentration of total carbohydrate was determined using the phenol-sulfate acid method [58]. The protein concentration was assessed by the Bradford method with bovine serum albumin as a standard [59]. The uronic acid content was determined by the sulfuric acid-carbazole method with galacturonic acid as standard [60]. The reducing sugar content was measured by the dinitrosalicylic acid method with glucose as standard [61]. The sulfate concentration was measured by the barium chloride-gelatin method with potassium sulfate as standard [62]. The total polyphenol content was determined using the Folin–Ciocalteu assay using gallic acid as a standard [63].

### 4.6. Molecular Weight

A HPGPC system (UltiMate 3000, Thermo Fisher Scientific, Waltham, MA, USA) combined with a multi-angle laser light scattering detector (DAWN HELEOS II, Wyatt technology, Santa Barbara, CA, USA) and a differential refractive index detector (Optilab T-rEX, Wyatt technology, Santa Barbara, CA, USA) was employed to determine the molecular weight. Both gel permeation columns, OHpak SB-805 HQ (300 × 8 mm, 13 μm) and SB-803 HQ (300 × 8 mm, 6 μm) (Showa Denko Co., Ltd., Tokyo, Japan), were equipped. A total of 1 mg of EPS was dissolved in 1 mL of 0.1 mol/L NaNO_3_ and filtered through a 0.22 μm filter before analysis. Finally, a 20 μL sample was isocratically eluted with a 0.1 mol/L NaNO_3_ (containing 0.02% NaN_3_) mobile phase at 0.8 mL/min.

### 4.7. Monosaccharide Compositions

A reverse-phase HPLC system (LC-20ADXR, Shimadzu, Tokyo, Japan) fitted with a Shim-pack GIST C18 Column (250 × 4.6 mm, 5 μm) (Shimadzu, Tokyo, Japan) and an ultraviolet monitor (SPD-20A, Shimadzu, Tokyo, Japan) set at 254 nm was used to analyze the monosaccharide compositions [64]. In brief, 10 mg of EPS was mixed with 5 mL of 2 mol/L trifluoroacetic acid (TFA), filled with nitrogen, and reacted at 105 °C for 6 h. The hydrolysate was dried with nitrogen, added with methanol, and evaporated with a rotary evaporator 5 times to remove the TFA. The EPS residue was dissolved in distilled water. EPS samples, nine monosaccharide standards (glucose, Glc; rhamnose, Rha; galactose, Gla; mannose, Man; arabinose, Ara; xylose, Xyl; glucuronic acid, GlcUA; galacturonic acid, GlaUA; and glucosamine, GlcN), and 0.05–0.5 mg/mL mixed monosaccharide standards were respectively added to 0.5 mol/L PMP methanol solution and 0.3 mol/L NaOH solution, and incubated at 65 °C for 1 h. For the neutralization of the reaction, 0.3 mol/L hydrochloric acid was blended into the reaction solution after it was cooled to room temperature. PMP was extracted three times by adding trichloromethane to the mixture and then centrifuging it at 12,000 rpm for 15 min to obtain the supernatant. Before being subjected to HPLC, the supernatant was filtered using a 0.22 μm filter. The mobile phase consisted of 0.1 mol/L phosphate buffer (pH = 6.7) and acetonitrile (83:17, *v*/*v*). The injection volume was set as 20 μL, and the flow rate was 0.8 mL/min.

### 4.8. FTIR Spectrum

FTIR was conducted to ascertain the functional groups of EPS. After mixing the lyophilized EPS and KBr in a ratio of 1:100, the mixture was compressed into a thin slice. Then, an FTIR spectrometer (Nicolet IS10, Thermo Fisher Scientific, Waltham, MA, USA) was used to scan the slice between 4000 and 400 cm^−1^.

### 4.9. Surface Morphology

Five milligrams of lyophilized EPS were glued to the sample stage with conductive adhesive and then coated with gold in a sputter coater (JFC-1100, JEOL, Tokyo, Japan). The surface morphology of the EPS was viewed by cold field emission scanning electron microscope (Hitachi SU8000, Tokyo, Japan) at various magnifications of 300× and 5000×. The measured accelerating voltage was set to 3 kV.

### 4.10. Thermogravimetric and Differential Scanning Calorimetric Analysis

The thermogravimetric properties of the EPS were analyzed using a TG-DSC 3+ thermogravimetric analyzer (Mettler Toledo, Zurich, Switzerland). The ground EPS (10 mg) was placed into an aluminum crucible and heated from 30 °C to 600 °C at a rate of 10 °C/min in a nitrogen atmosphere.

### 4.11. Antioxidant Capacity of EPS

#### 4.11.1. ABTS Radical Scavenging Assay

The ABTS radical scavenging capacity of EPS was evaluated in line with the method described by Wang et al. [11] with some modifications. The ABTS (7 mmol/L) and potassium persulfate (2.45 mmol/L) were blended thoroughly in an equal volume and reacted overnight at room temperature in the dark to generate ABTS radicals. Subsequently, 600 μL ABTS solution was mixed with 300 μL of EPS at various concentrations (0.5–10 mg/mL), respectively, and the solution was reacted for 10 min. The reaction solution was detected at 734 nm. As a positive control, VC at an equal concentration was employed. The ABTS scavenging rate of EPS was calculated according to the following equation:

(3) $$\mathrm{ABTS}\;\mathrm{scavenging}\;\mathrm{rate}\;(\%)=(1\;-\;\frac{A_{\mathrm{sample}}-A_{\mathrm{blank}}}{A_{\mathrm{control}}})\;\times \;100\%$$

The absorbance of the EPS group (containing EPS and ABTS) was set as A_sample_, the absorbance of the negative control group (containing distilled water and ABTS) was set as A_control_, and the absorbance of the blank group (containing EPS and distilled water) was set as A_blank_.

#### 4.11.2. DPPH Radical Scavenging Assay

An adjusted protocol was performed to assess the scavenging capacity of DPPH radical [65]. An aliquot of EPS (0.5–10 mg/mL) or VC (0.5–10 mg/mL) was mixed with an equal volume of 0.2 mmol/L DPPH solution (prepared in methanol) and kept at room temperature for half an hour. The mixture was examined at 517 nm. The following equation was used to calculate the DPPH scavenging activity of EPS.

(4) $$\mathrm{DPPH}\;\mathrm{scavenging}\;\mathrm{rate}\;(\%)=(1\;-\;\frac{A_{\mathrm{sample}}\;-\;A_{\mathrm{blank}}}{A_{\mathrm{control}}})\;\times \;100\%$$

The absorbance of the EPS group (EPS and DPPH) was denoted as A_sample_, negative control (distilled water and DPPH) as A_control_, and the absorbance of the blank group (EPS and methanol) as A_blank_.

### 4.12. Anti-Inflammatory Activity of EPS

#### 4.12.1. Cell Culture

The RAW264.7 cells were cultured in DMEM supplemented with 10% FBS, 100 U/mL penicillin, and 100 μg/mL streptomycin. The cells were grown at 37 °C in a humidified atmosphere containing 5% CO_2_ in an incubator (Forma 3, Thermo Fisher Scientific, Waltham, MA, USA).

#### 4.12.2. Cell Viability of EPS

The effect of EPS on cell viability was evaluated by the cell counting kit-8 (CCK-8). RAW264.7 cells (1.0 × 10^4^ cells per well) were inoculated in 96-well plates and cultured overnight. Then, lyophilized EPS was diluted with DMEM at a final concentration of 0, 10, 20, 30, 40, 50, 100, 200, and 500 μg/mL and administered to RAW264.7 cells for 24 h. After incubation, DMEM and CCK-8 solution were mixed in a ratio of 10:1, applied to the well, and incubated for 2 h at 37 °C in the dark. The supernatant’s absorbance was examined at 450 nm. The cell viability was calculated using the equation:

(5) $$\mathrm{Cell}\;\mathrm{viability}\;(\%)=\frac{A_{\mathrm{sample}}\;-\;A_{\mathrm{blank}}}{A_{\mathrm{control}}\;-\;A_{\mathrm{blank}}}\;\times \;100\%$$

where A_sample_ represented the absorbance of EPS-treated cells and CCK-8, A_blank_ represented the absorbance of DMEM and CCK-8, and A_control_ represented the absorbance of EPS-untreated cells and CCK-8.

#### 4.12.3. Establishment of LPS-Induced Inflammatory Model

LPS from *Escherichia coli* O111: B4 was used to construct a macrophage inflammatory model. Briefly, 1.0 × 10^4^ RAW264.7 cells were incubated in 96-well plates for 12 h. In addition, cells were subjected to 50, 100, and 500 μg/mL EPS and cultured for 12 h. Subsequently, 1 μg/mL LPS stimulated the cells for 24 h.

To determine whether the model was successfully constructed, the Griess method was used to measure the level of the inflammatory mediator NO [66]. An equal volume of Griess A and Griess B was successively mixed with the supernatant, and then the absorbance of the solution was measured at 540 nm. The NO content was calculated using NaNO_2_ as a standard.

#### 4.12.4. RNA Extraction and Quantitative Real-Time Polymerase Chain Reaction

RAW264.7 cells were seeded at a density of 2.0 × 10^6^ cells per well into 6-well plates, followed by induction of the inflammatory model as previously described. Total RNA was isolated from the RAW264.7 using TRIzol reagent following the protocol of the manufacturer. Agarose gel electrophoresis and NanoDrop were conducted to detect the RNA integrity and purity, respectively. The total RNA was used for reverse transcription of cDNA by the M5 Super plus qPCR RT kit (Mei5 Biotechnology Co., Ltd., Beijing, China). qPCR was conducted using M5 HiPer SYBR Premix EsTaq (Mei5 Biotechnology Co., Ltd., Beijing, China). Table S1 presents the details of the primers. Two-step amplification reactions were used in this study: 1 cycle of 95 °C for 30 s, 40 cycles of 95 °C for 10 s and 60 °C for 30 s, and then 1 cycle of 95 °C for 5 s, 60 °C for 60 s, and 95 °C for 1 s for the melting curve. The data were analyzed according to the 2^−ΔΔCt^ method with glyceraldehyde-3-phosphate dehydrogenase as an internal reference [67].

### 4.13. Statistical Analysis

The data are presented using the mean values ± standard error of the mean (SEM). Each experiment was repeated three times, except for the cell viability and NO detection assays, which were six replicates. Multiple comparisons using Tukey’s test were conducted after the differences between the groups were examined by one-way analysis of variance (ANOVA) in SPSS software 26.0. Statistical significances were represented as *p* < 0.05.

## 5. Conclusions

In conclusion, we investigated the optimization strategy and biological functions of a novel postbiotic-crude EPS isolated from *Bacillus subtilis* H4 in our laboratory. Systematic optimization of *Bacillus subtilis* H4 fermentation conditions through a four-step strategy resulted in a significant increase in EPS yield to 15.01 g/L. EPS is a large molecular weight-neutral heteropolysaccharide composed of galactose, glucose, glucosamine, arabinose, and mannose. This EPS has excellent thermostability, antioxidant, and anti-inflammatory activity. These findings provide insight into the biological functions of EPS, expand our understanding of efficient EPS production strategies, and suggest new opportunities for the development of EPS as a novel functional additive in the food industry.

## Figures and Tables

**Table 1:** Analysis of variance of factors affecting EPS yield.

Factors	Sum of Squares	df ^1^	Mean Square	Coefficient Estimate	*F*-Value	*p*-Value ^2^
Model	30.89	7	4.41		8.16	0.030 *
A-Sucrose	4.22	1	4.22	0.59	7.80	0.049 *
B-Soy peptone	10.40	1	10.40	0.93	19.23	0.012 *
C-MnSO_4_	12.42	1	12.42	1.02	22.96	0.009 **
D-MgSO_4_	0.023	1	0.02	0.04	0.04	0.847
E-Inoculum concentration	1.40	1	1.40	−0.34	2.58	0.183
F-Temperature	0.95	1	0.95	−0.28	1.76	0.255
G-pH	1.48	1	1.48	−0.35	2.74	0.173

R^2^ = 0.9345,^1^ df: degree of freedom; ^2^ * *p* < 0.05, ** *p* < 0.01.

**Table 2:** Experimental design and results of Box–Behnken.

Run	Level (%)			EPS Yield (g/L)	
	X_1_-Soy Peptone	X_2_-Sucrose	X_3_-MnSO_4_	Experimental Value	Predicted Value
1	0	0	0	13.41	14.07
2	−1	−1	0	5.80	5.63
3	0	−1	−1	6.16	5.42
4	1	0	1	11.00	10.09
5	−1	0	1	12.71	11.99
6	0	1	1	11.87	12.61
7	1	−1	0	8.07	8.09
8	1	1	0	6.86	7.03
9	−1	1	0	11.47	11.45
10	0	0	0	14.75	14.07
11	0	0	0	13.36	14.07
12	0	−1	1	6.86	7.74
13	0	0	0	14.03	14.07
14	1	0	−1	5.48	6.20
15	−1	0	−1	5.37	6.27
16	0	1	−1	6.20	5.32
17	0	0	0	14.13	14.07

**Table 3:** ANOVA analysis of response surface quadratic model.

Factors	Sum of Squares	df ^1^	Mean Square	*F*-Value	*p*-Value ^2^
Model	203.12	9	22.57	23.63	<0.001 ***
X_1_	1.94	1	1.94	2.03	0.198
X_2_	11.31	1	11.31	11.84	0.011 *
X_3_	46.15	1	46.15	48.32	<0.001 ***
X_1_X_2_	11.84	1	11.84	12.40	0.010 **
X_1_X_3_	0.83	1	0.83	0.87	0.382
X_2_X_3_	6.17	1	6.17	6.46	0.039 *
X_1_^2^	27.92	1	27.92	29.23	0.001 ***
X_2_^2^	49.80	1	49.80	52.14	<0.001 ***
X_3_^2^	34.28	1	34.28	35.89	<0.001 ***
Residual	6.69	7	0.96		
Lack of fit	5.38	3	1.79	5.48	0.067
Pure error	1.31	4	0.33		
Cor total	209.80	16			

R^2^ = 0.9681, ^1^ df: degree of freedom; ^2^ * *p* < 0.05, ** *p* < 0.01, *** *p* < 0.001.

**Table 4:** Chemical composition of EPS.

Sample	Component (%)				
	Total Carbohydrate	Protein	Uronic Acid	Reducing Sugar	Sulfate
EPS	85.37	0.25	ND ^1^	ND	ND

^1^ ND: not detected.

**Table 8:** Factors and corresponding levels of Box–Behnken experiment.

Factors	Level (%)		
	−1	0	+1
X_1_-Soy peptone	3.0	4.5	6.0
X_2_-Sucrose	7.0	8.5	10.0
X_3_-MnSO_4_	0.040	0.055	0.070

## Data Availability

The data presented in this study are available on request from the corresponding author.

## Supplementary Materials

The following supporting information can be downloaded at: https://www.mdpi.com/article/10.3390/molecules30102089/s1, Table S1: Primer sequences used for qRT-PCR in this study.

## Abbreviations

The following abbreviations are used in this manuscript:

ISAPP	International Scientific Association of Probiotics and Prebiotics
EPS	Exopolysaccharide
ROS	Reactive oxygen species
PBD	Plackett–Burman design
RSM	Response surface methodology
TG	Thermogravimetric
DTG	Differential thermogravimetric
DSC	Differential scanning calorimetry
VC	Ascorbic acid
LPS	Lipopolysaccharide
NO	Nitric oxide
PSA	Path of steepest ascent
BBD	Box–Behnken design
BPY	Beef peptone yeast
ABTS	2,2′-azino-bis-3-ethylbenzothiazoline-6-sulfonic acid
DPPH	1,1-diphenyl-2-picrylhydrazyl
DMEM	Dulbecco’s Modified Eagle Medium
FBS	Fetal bovine serum
HPGPC	High-performance gel permeation chromatography
HPLC	High-performance liquid chromatography
Mw	Weight-average molecular weight
Mn	Number-average molecular weight
FTIR	Fourier Transform infrared spectroscopy
PMP	1-phenyl-3-methyl-5-pyrazolone
SEM	Standard error of the mean
CCK8	Cell counting kit-8
ANOVA	Analysis of variance
