# Supplementary material for: Efficient Production Strategy of a Novel Postbiotic Produced by Bacillus subtilis and Its Antioxidant and Anti-Inflammatory Effects
Source: Molecules. 2025 May 8;30(10):2089. doi: 10.3390/molecules30102089 (PMC12113817; doi:10.3390/molecules30102089)
Supplement: Supplementary file 1 [file molecules-30-02089-s001.zip › molecules-3589741-supplementary.pdf]

Supplementary materials

Table S1. Primer sequences used for qRT-PCR in this study.

Gene	Forward/Reverse	Squence (5'-3')	Accession number	Product length
iNOS	F	CAGCGGAGTGACGGCAAACAT	AY090567.1	184
	R	GCAAGACCAGAGGCAGCACATC		
COX2	F	ATCAGGTCATTGGTGGAGAGGTGTAT	NM_011198.4	237
	R	TGCTGGTTTGGAATAGTTGCTCATCA		
IL-1β	F	AATCTCGCAGCAGCACATCAACA	NM_008361.3	197
	R	ACACCAGCAGGTTATCATCATCATCC		
IL-6	F	GCTACCTGGAGTACATGAAGAACAACTTA	NM_031168.2	216
	R	AGATGAATTGGATGGTCTTGGTCCTTAG		
TNF-α	F	GCCCAGACCCTCACACTCAGAT	NM_013693.2	220
	R	AGCCTTGTCCCTTGAAGAGAACCT		
TLR4	F	TGTGTCAGTGGTCAGTGTGATTGTG	NM_021297.2	225
	R	CTGTAGTGAAGGCAGAGGTGAAAGC		
MyD88	F	GACGATTATCTACAGAGCAAGGAATGTGA	NM_010851.3	151
	R	CGCATATAGTGATGAACCGCAGGAT		
NF-κB	F	GTCTTACACTTAGCCATCATCCACCTC	NM_008689.2	165
	R	ATCCTCTACTACATCTTCCTGCTTGGT		
GAPDH	F	GTGAAGGTCGGTGTGAACGGATT	GU214026.1	235
	R	GGTCTCGCTCCTGGAAGATGGT		
